# Guidelines to support HIV-affected individuals and couples to achieve pregnancy safely: Update 2018

**DOI:** 10.4102/sajhivmed.v19i1.915

**Published:** 2018-10-18

**Authors:** Natasha E.C.G. Davies, Gail Ashford, Linda-Gail Bekker, Nomathemba Chandiwana, Diane Cooper, Silker J. Dyer, Lauren Jankelowitz, Otty Mhlongo, Coceka N. Mnyani, Muhangwi B. Mulaudzi, Michelle Moorhouse, Landon Myer, Malika Patel, Melanie Pleaner, Tatiana Ramos, Helen Rees, Sheree Schwartz, Jenni Smit, Doreen S. van Zyl

**Affiliations:** 1 Wits Reproductive Health and HIV Institute, Faculty of Health Sciences, University of the Witwatersrand, South Africa; 2 Wits Donald Gordon Medical Centre, South Africa; 3 The Desmond Tutu HIV Centre, Institute of Infectious Disease and Molecular Medicine, University of Cape Town, South Africa; 4 Department of Medicine, Faculty of Health Sciences, University of Cape Town, South Africa; 5 School of Public Health, University of Western Cape, South Africa; 6 Department of Obstetrics and Gynaecology, Groote Schuur Hospital, Faculty of Health Sciences, University of Cape Town, South Africa; 7 Southern African HIV Clinicians’ Society, South Africa; 8 KwaZulu-Natal Department of Health, South Africa; 9 Department of Obstetrics and Gynaecology, School of Clinical Medicine, University of the Witwatersrand, South Africa; 10 Division of Epidemiology and Biostatistics, School of Public Health and Family Medicine, University of Cape Town, South Africa; 11 Department of Epidemiology, Johns Hopkins School of Public Health, United States; 12 Private practitioner, South Africa

## Foreword

In June 2011, the Southern African HIV Clinicians’ Society, together with an expert working group, developed the *Guidelines on Safer Conception in Fertile HIV-Infected Individuals and Couples*.^1^ Since then, interventions to manage and prevent HIV have evolved and, similarly, options for safer conception have expanded. These updated guidelines provide healthcare providers with up-to-date information to support efforts to optimise safer conception care.

## Key game changers between 2011 and 2018

- Safer conception has been incorporated into various national policies and strategies, including in South Africa, Kenya, Uganda and Botswana.^2,3,4,5^
- In 2016, universal test and treat (UTT) was adopted in South Africa and other southern African countries.^3,5,6^ This makes provision for antiretroviral therapy (ART) initiation when people test HIV-positive, regardless of their CD4+ count. UTT improves health outcomes and contributes to treatment as prevention.
- Mounting evidence indicates that an undetectable viral load (VL) means that a person is not infectious^7,8,9,10^ (with certain provisions, such as ongoing treatment adherence and absence of sexually transmitted infections [STIs]). This is a key game changer that emphasises the importance of any HIV-positive partner being on ART and virally suppressed before they start trying to achieve pregnancy.
- Since 2013, Option B+ has been rolled out in many countries to provide lifelong ART for all HIV-positive pregnant and breastfeeding women to prevent mother-to-child transmission (MTCT) of HIV.^11,12,13^
- Reassuring safety data are now available concerning ART exposure during pregnancy and breastfeeding, including efavirenz use.^14,15,16^
- However, a new safety alert has emerged for dolutegravir use in women around the time of conception, with an indication of increased risk of neural tube defects (NTDs). Further research is needed but, in the interim, providers are recommended to avoid the use of dolutegravir in any woman who expresses a desire for pregnancy now or in the near future and to counsel all women of childbearing potential about the possible risks of becoming pregnant while taking dolutegravir. Providers should adhere to World Health Organization (WHO) or relevant local recommendations as they evolve.
- Tenofovir/emtricitabine (Truvada^®^ and equivalent generics) has been registered for use as pre-exposure prophylaxis (PrEP) for HIV prevention among individuals at high risk of HIV infection by the National Regulatory Authorities of Kenya, Lesotho, Malawi, Namibia, South Africa, Zambia and Zimbabwe.^17^ Approval is pending in Botswana, Nigeria and Uganda, although their national health policies incorporate PrEP as a key prevention intervention.^17^
- The WHO recently recommended that tenofovir-based PrEP should be continued for pregnant and breastfeeding women who *remain at substantial risk of HIV acquisition* during this period. This recommendation is accompanied by an emphasis on continued pharmacovigilance for any adverse maternal or infant outcomes.^18^
- The combination of ART initiation in the HIV-positive partner with PrEP coverage in the HIV-negative partner until the positive partner is confirmed virally suppressed (VL < 200 copies/mL) and has been on treatment for at least six months has been shown to completely remove the risk of HIV transmission where adherence to both ART and PrEP is maintained.^19^
- Where the HIV-positive partner(s) are virally suppressed, then all other safer conception strategies become optional. Couples may, however, still choose to use other options, so the full range of strategies should still be offered to all HIV-affected couples to enable their informed choice.^20^
- For the purposes of this guideline, an undetectable VL is considered any VL that is 200 copies/mL or less to allow for variable sensitivity of viral load assays across the region.^7^
- Increasing evidence shows that there is little to be gained by offering other assisted reproductive technologies and/or sperm washing to avoid HIV transmission, if the HIV-positive partner is adhering to ART and is confirmed to be virally suppressed.^21^
- It is no longer considered necessary to refer couples with presumed normal fertility for assisted reproductive technologies such as sperm washing and intrauterine insemination (IUI) unless this is their choice and they are made aware of the costs involved.
- There is wider availability and access to long-acting reversible contraceptives, such as the subdermal contraceptive implant and the intrauterine contraceptive device (IUCD) – relevant because both allow immediate return to fertility on removal, unlike predominantly used hormonal injectables, which have a longer period for return to fertility. There are some drug interactions with ART, which are discussed in more detail below.^22^

## Introduction

In southern and sub-Saharan Africa, the majority of HIV-positive individuals are adults of reproductive age,^23^ many of whom desire children^24,25,26,27,28^ and between 30% and 50% of them have an HIV-negative partner,^29,30^ although many may remain unaware of their own or their partner’s status.^31,32,33^ Attempting natural conception, without using any HIV risk reduction strategies, places HIV-affected couples at increased risk of HIV transmission.^28^ This likely contributes to regional estimates that up to 60% of new HIV infections occur in stable serodifferent couples.^34^ Acknowledging the considerable overlaps between high HIV prevalence in reproductive-aged individuals, HIV transmission risks and active fertility desires, safer conception services need to be scaled up across the region.^35,36^

Modern ART, which is highly effective, with lower toxicities and pill burden, ensures that HIV can be managed as a lifelong, chronic disease and that MTCT and infection of an HIV-negative partner can be almost entirely avoided if testing and treatment are effectively offered and utilised. With this in mind, the medical and ethical arguments previously used to deny the rights of HIV-positive women to become pregnant, or HIV-positive men to have biological children, are becoming increasingly irrelevant.^33^ Healthcare providers need to actively engage with people living with HIV (PLHIV), and their partners, to respect and support the fulfilment of their reproductive rights and desires. Parenting should now be normalised for any individual or couple affected by HIV. These guidelines attempt to provide practical information about how this goal can be achieved safely, with optimal health and minimal HIV acquisition risks for any uninfected partner and the resulting child.

The routine provision of appropriate contraception and safe prepregnancy planning support holds the potential to impact adult HIV testing and ART uptake, VL outcomes, adult HIV prevention efforts and the achievement of elimination of mother-to-child transmission (EMTCT).^35^ It is one of the few cross-cutting interventions that can positively impact across numerous HIV and sexual and reproductive health programmes to the benefit of women, men and children. This requires accessible and safe fertility planning services and includes both the prevention of unplanned pregnancy through the use of appropriate contraceptive methods and the achievement of a planned, healthy pregnancy.

### Scope of the guidelines

The guidelines are designed to assist healthcare providers to, first, identify clients’ fertility desires and second, provide safe and effective pregnancy planning guidance to a presumed fertile couple who currently desire a child and where one or both partners are known to be living with HIV or are at possible risk of acquiring HIV because of having condomless sex with a partner(s) of unknown HIV status.

Both resource-limited settings, such as most public health sector facilities across the region, and resource-intensive clinical settings, including the private sector, where assisted reproductive technologies may be available, have been considered. However, even in resource-intensive settings, with effective ART and other risk reduction strategies, costlier assisted reproductive technology, for example, sperm washing, IUI and *in vitro* fertilisation (IVF), should no longer be considered necessary purely for the prevention of HIV transmission.^20,21,35^

It is important to recognise infertility and refer individuals or couples who are unable to achieve pregnancy; however these guidelines do not cover the management of infertility.

It should be noted that much of what is covered in these guidelines is relevant to all couples desiring pregnancy.

### Structure of the guidelines

The guidelines are presented in four sections.

The first section, ‘Discussing fertility and childbearing with HIV-affected women and men’, discusses how providers can raise the issue of fertility planning and help identify the desires of HIV-affected women and men in relation to preventing an unwanted pregnancy or planning a desired pregnancy.

The second section, ‘HIV-affected clients who express a desire to have a child’, focuses on the management of individuals and couples who desire pregnancy, with an emphasis on management of HIV and other comorbidities prior to attempting pregnancy. This section includes strategies for serodifferent and seroconcordant couples, as well as undisclosed individuals or individuals with an unknown status partner, to minimise risks of horizontal and vertical HIV transmission.

The third section, ‘Clients with no immediate plans for a child’, provides a very brief overview of contraceptive provision within the context of HIV for those who do not desire a child at present or in the future.

The fourth section, ‘Additional considerations for optimising prepregnancy health’, provides an overview of several additional service delivery issues, including prepregnancy counselling, basic assessment for underlying infertility, as well as management of comorbidities, pregnancy in older women and basic management if pregnancy is confirmed or a miscarriage occurs. Special considerations for the provision of counselling for HIV-affected single women, same sex and transgender individuals and couples desiring pregnancy are briefly covered.

### A word on terminology used in the guidelines

#### Safer conception

*Safer conception* is the term used throughout these guidelines to refer to the overall process of choosing one or more risk reduction strategies to try and minimise HIV transmission and acquisition risks while attempting to achieve a healthy pregnancy. Although in the field of fertility medicine the word *conception* is no longer used because it is not a distinct biological event,^37^ for the purposes of these guidelines it was felt important to maintain continuity with existing literature and guidelines, which speak to safer conception with particular reference to minimising HIV risks during the time when an HIV-affected couple is attempting pregnancy.

#### HIV-affected individuals and couples

The term *HIV-affected individuals and couples* includes a range of HIV relationship combinations, all of which require specifically tailored support to minimise HIV risk and to maximise the possibility of a safe pregnancy. Couples may be in a mutually disclosed seroconcordant or serodifferent relationship, relationships where one or both partners have unknown HIV status or partnerships in which one individual is living with HIV and feels unable to disclose their status to their partner. HIV-positive and -negative individuals without a stable partner may also desire a child. HIV-affected men who have sex with men, women who have sex with women, bisexual and transgender individuals and couples may also have reproductive desires requiring the support of healthcare providers. In high HIV prevalence settings all HIV-negative individuals who have a known positive or unknown status partner fall within this definition of ‘HIV-affected’ and should be offered safer conception services if they desire a child.

#### Safe pregnancy

Safe or healthy pregnancy in this case refers to a minimised risk of HIV transmission to an uninfected partner and/or a foetus.

#### Horizontal HIV transmission

The transmission of HIV from one sexual partner to another is called *horizontal transmission*. It can also occur between people who inject drugs if they share injecting equipment.

#### Vertical HIV transmission

Vertical transmission is the transmission of HIV from mother-to-child, either during pregnancy, labour and delivery or breastfeeding.

#### Condomless sex

These guidelines specifically use the term *condomless sex* as opposed to *unprotected sex* because, with the availability of ART and PrEP, when a couple undertake sexual intercourse without a condom they are not unprotected; they are simply using other measures to protect themselves and their partner from HIV transmission and acquisition. This distinction is important because the phrase *unprotected* or *unsafe* sex creates anxiety in couples and also implies unsafe and irresponsible behaviour when, in fact, many of these couples are very committed to doing everything possible to achieve pregnancy safely and reduce risk to the HIV-negative partner.

#### Timed condomless sex

Timed condomless sex is one safer conception strategy that individuals or couples may choose to use to reduce the total number of HIV risk exposures undertaken while trying to achieve pregnancy. With this strategy, condomless sex acts are limited to the peak fertile window, which occurs around the time that the female partner ovulates. This strategy has also been referred to as *timed periovulatory unprotected intercourse*.

#### Healthcare provider(s)

This term refers to all clinicians, including doctors and nurses, and other staff such as counsellors and community healthcare workers who provide contraception, fertility planning and prepregnancy services for people affected by HIV, as defined by their scope of practice.

#### Serodifferent (serodiscordant)

The guidelines use the term *serodifferent* instead of *serodiscordant* to describe relationships in which one partner is HIV-positive and the other is HIV-negative. Other terms with the same meaning are *seromixed, mixed status* or *magnetic couples*. The term serodifferent is used in these guidelines rather than *serodiscordant* in response to the expressed preferences of PLHIV and their partners.

#### Disclosure and partial disclosure

In the context of these guidelines, *disclosure* is the process of revealing one’s HIV status to another person. Disclosure may also involve telling another person about his or her HIV treatment, health status or VL.

Partial disclosure, for the purposes of this document, refers to a situation in which one partner has disclosed their HIV status but may not have told the other partner all of the details of their HIV history. This situation can commonly arise when an individual who already knows his or her status, and may be well established on ARVs, chooses to disclose by attending for couples testing and counselling with their partner as if testing for the first time.

Partial disclosure may also refer to other aspects of a person’s history including previous pregnancies, children, miscarriages, terminations of pregnancy or failure to conceive with other partners.

#### Undetectable = uninfectious (U=U)

U=U is an abbreviation for *undetectable = uninfectious* or *untransmissible*. Over time, evidence has shown that if an HIV-positive person is well established on ART, adherent to treatment and has a recently confirmed, undetectable plasma VL, this person is considered uninfectious and will not transmit HIV (untransmissible).^8,9,10^ This is discussed further in Box 2.

#### Infertility

Infertility is defined as the failure to establish a clinical pregnancy after 12 months or more of regular, condomless sexual intercourse in a non-contracepting couple. Infertility may be a result of an impairment of a person’s capacity to reproduce either as an individual or with his or her partner and can be a result of female factors, male factors or a combination of both.^37^

## Discussing fertility and childbearing with HIV-affected women and men

Despite improved quality of life and normal life expectancy resulting from effective ART, many HIV-positive and HIV-affected clients remain reluctant to discuss sexual activity, fertility and childbearing with their healthcare providers.^25,26,33^ It is important for providers to provide culturally sensitive prepregnancy counselling (see Appendix 1) and reproductive health services.^2,27^ Providers should be able to confidently initiate the discussion, create a conducive environment for open discussion and be able to provide fertility planning services tailored to the individual’s or couple’s goals and choices (section ‘Important points to note when discussing fertility choices and desires’, Figure 1 and Table 1).

**FIGURE 1:**
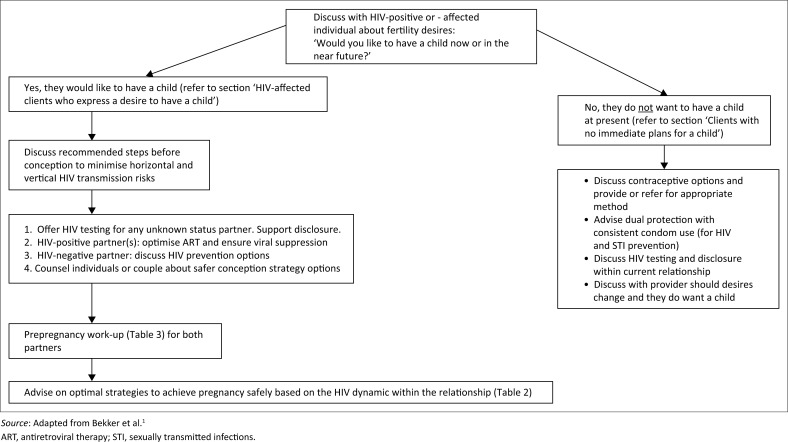
Approach to discussing fertility desires with HIV-affected individuals and couples.

**TABLE 1:** Additional factors to consider when discussing fertility intentions.

Discuss health-related influences	Discuss options – where relevant	Discuss personal circumstances
Client’s health status especially in relation to achieving and maintaining pregnancy. Age – risks associated with advanced maternal age in women > 35 years (see section ‘Identifying and managing comorbidities’). Previous attempts to achieve pregnancy. How long did it take to achieve pregnancy? Any previous pregnancy losses or terminations of pregnancy? Possibility of HIV transmission to partner and baby including importance of ART adherence and sustained viral suppression. Current method of contraception, and implications for return to fertility (injectable may take 6–9 months, all other methods, immediately after stopping or removing the method).	Explore alternative options for having a child other than achieving pregnancy, especially if client’s history suggests underlying infertility, if it is a same-sex couple or a single person. Can include fostering, adoption and informally caring for a relative’s child.	Current and desired number of children? Number of children clients have with previous and current partners, including age, general health status? HIV status of children and whether well on ART? Previous child bereavement(s)? Current and future resources available to care for a child? Eligibility for childcare grant. Relationship with current partner – does the other partner also want a child? Partner, family and community influences on fertility desires?

ART, antiretroviral therapy.

### Important points to note when discussing fertility choices and desires

- Normalise the discussion:
  ■ People may feel uncomfortable discussing sex, including their desire to prevent or plan for pregnancy, when one or both partners are HIV-positive. It is important to normalise this discussion, creating an honest and open atmosphere. ‘Normalising’ the discussion includes pointing out that many other PLHIV and uninfected people are grappling with these issues, and many have conceived. This communicates that the healthcare provider is non-judgemental and supportive of reproductive rights.
  ■ Frequent discussions about fertility choices make this a normal part of a consultation and acknowledge that fertility desires change over time.
- Offer HIV testing:
  ■ HIV testing and counselling for anyone with unknown HIV status (or repeat testing for those previously testing HIV-negative) should be revisited regularly over time.
- Focus on both partners and be sensitive to dynamics within the relationship:
  ■ Discussions about fertility planning (both the desire to achieve pregnancy or to prevent pregnancy) often focus on female clients. However, it is important to engage male clients as many men living with HIV wish to have children.^38^ In some relationships, the male in the partnership has a strong influence over women’s fertility-related desires and decisions, often determining when the next child will be planned or whether a reliable form of contraception will be used.^39,40^
  ■ Couple-based HIV testing and counselling should be routinely encouraged. Efforts should be made to determine the HIV status of both partners; however, coercion and undue pressure should be avoided.
  ■ Although there are several advantages of involving partners, the provider needs to be sensitive to the fact that some women may not be able to convince their partner to come in; the provider should let the client make this decision.
  ■ Providers should be sensitive to issues relating to gender inequality and gender-based violence within relationships. It is important to provide a platform where both partners can agree on their desire to plan for, or prevent pregnancies, without any pressure being exerted on an unwilling partner. It can be helpful to have these discussions with each partner separately, as well as together, if possible, to ascertain if such imbalances are present.
- Encourage informed decision-making:
  ■ An important part of these discussions should be to ensure that all clients have a basic understanding of the menstrual cycle, HIV transmission, HIV prevention options and how to achieve pregnancy safely with reduced risks of horizontal and vertical HIV transmission, as well as how to prevent unplanned pregnancy for those not currently desiring pregnancy. The uptake of contraceptive and safer conception services should be promoted as appropriate.
  ■ The provider should seek to assist clients to arrive at their own informed choice about their fertility desires. Couples who are unsure of their fertility intentions should be encouraged and supported to access shorter-term contraception while they formulate a decision and discuss their plans during follow-up consultations.
  ■ Providers should be trained to offer a range of effective prevention alternatives, including viral suppression with ART and PrEP.
- Counselling about fertility intentions is not a once-off event:
  ■ Providers should reassure clients that fertility intentions can change over time, and that they are welcome to revisit their decision and discuss any changes in the future. Providers should raise the issue of fertility intentions regularly and not rely on the client to do so.^33^ Documenting decisions ensures continuity of care, particularly in busy public sector clinics where clients may be managed by different providers.

### Concluding the discussion – Different outcomes require different approaches

- **Individuals or couples who express a desire for pregnancy** (see the section ‘HIV-affected clients who express a desire to have a child’) should be supported in starting the process of achieving pregnancy safely, utilising appropriate risk reduction strategies to minimise horizontal and vertical HIV transmission or acquisition risks. Whatever the client’s expressed desire, he or she should be encouraged, where possible, to return to the facility with his or her partner to enhance the provision of couple-based fertility counselling and services. However, this should not be a prerequisite for future access to services.
- **Individuals and couples who do not currently desire a pregnancy** (see the section ‘Clients with no immediate plans for a child’) should be provided with information about available contraceptive methods, HIV testing and effective HIV prevention options or HIV treatment. They should be encouraged to discuss any changes in their plans over time with their provider. Providers should also regularly revisit this discussion.
- **Individuals and couples who are not yet sure** of their fertility desires may require further counselling and shorter-term contraception, and the issue should be revisited again in the near future.

### Working with couples

- Clients often attend consultations on their own. Discussions regarding fertility and planning for pregnancy should be undertaken as appropriate – some clients may prefer one-on-one to couple-based sessions. There are several advantages to a couple-based approach as outlined in Box 1.

BOX 1 Advantages to a couple-based approach.

- Partners may have similar or different expectations and needs.
  ■ They may never have had an open discussion with each other about this before – respective views will have an important influence on fertility decisions.
  ■ This approach assists couples to arrive at appropriately informed decisions about their mutually agreed upon fertility choices.
- The health of both partners is important and should be optimised as part of achieving pregnancy safely.
- Where a couple encounters difficulty in achieving pregnancy, it is important to involve both partners. Where possible, both partners should be referred for infertility investigations and interventions.

### Working with couples and disclosure

Engaging both partners together requires full HIV status disclosure. This can raise many issues about which a provider should be sensitive:

- Where full disclosure has not yet happened, providers should consider the possibility of partial disclosure. Partners should be consulted separately at least once to explore any undisclosed issues about which the healthcare provider should be aware. This may include HIV history (time of diagnosis and treatment initiation) and any previous terminations of pregnancy, miscarriages or failure to achieve pregnancy, with other partners. The provider should discuss with each partner individually what aspects may be relevant to disclose to their partner.
- In some situations, disclosure of HIV status is not possible and disclosure should not be considered a prerequisite of safer conception care and support. Disclosure can be associated with risks, including intimate partner violence (particularly for women) and relationship breakdown, with loss of economic support.^32,41^ These issues need to be taken into account when encouraging disclosure. Forced disclosure, or a judgmental approach towards non-disclosure, may cause the undisclosed individual to withdraw from care altogether, placing them and their partner at even higher risk of HIV transmission and poor health outcomes. Risks can still be mitigated even where disclosure has not occurred and the individual who has not disclosed should be optimally managed within this context.
- Couple-based HIV testing and counselling may represent a valuable opportunity to assist with supported disclosure, whereby the known positive individual tests with their partner as if for the first time. However, the resulting partial disclosure should be carefully noted to avoid inadvertent discussion of as yet undisclosed details about previous illnesses or ARV treatment history to the other partner.
- Despite the importance of a couple-based approach, it is not uncommon for providers to be approached by individuals who desire a child but either do not know the serostatus of their partner, may not have a regular partner or may have a partner who is not willing to engage in care. Providers should develop an approach that ensures these individuals are also able to achieve pregnancy as safely as possible in a non-judgemental, supportive environment (see section ‘Integrated package of care’, Table 3).
- Other issues relating to disclosure include previous pregnancies, miscarriages, abortions or difficulties in getting pregnant with other partners. These can be discussed with the client and support provided in his or her decision as to how much he or she chooses to disclose to his or her current partner.

## HIV-affected clients who express a desire to have a child

Between 30% and 50% of HIV-positive individuals desire a child.^24,25,26,27^ Many may already be actively trying to achieve pregnancy at the time of clinical consultation. In supporting these individuals, there are a number of considerations that need to be taken into account.

These guidelines focus on low-cost, low-technology strategies. For those clients who can access more resource-intensive options, such as sperm-washing with IUI, IVF or intracytoplasmic sperm injection (ICSI), these may also be considered. However, where there are no concerns about infertility, these interventions may not add any additional benefit, add extra cost and unnecessarily medicalise what can now be a safe, natural process.^21,35^ Where available, these assisted reproductive technologies may provide additional options for couples who have suspected or confirmed underlying infertility. Identifying such couples is discussed briefly in the fourth section, ‘Additional considerations for optimising prepregnancy health’.

UTT and improved access to ART provide an important option for HIV prevention in serodifferent couples – as explained in Box 2.

BOX 2 The concept of U=U.

In the era of effective ART, the most effective strategy is to ensure that any HIV-positive partner is established on ART, adherent to treatment and has a recently confirmed undetectable plasma viral load (VL). In this situation, the person is considered U=U, meaning undetectable = uninfectious or untransmissible.

**U=U is further explained as follows:**

‘When ART results in viral suppression, defined as less than 200 copies/mL or undetectable levels, it prevents sexual HIV transmission. Across three different studies, including thousands of couples and many thousand acts of sex without a condom or pre-exposure prophylaxis (PrEP), no HIV transmissions to an HIV-negative partner were observed when the HIV-positive person was virally suppressed. This means that people who take ART daily as prescribed and achieve and maintain an undetectable viral load have effectively no risk of sexually transmitting the virus to an HIV-negative partner’.

U.S. Centers for Disease Control & Prevention, Dear Colleague Letter (September, 2017)^42^

**What is an undetectable viral load?**

Results of key studies conclude that people with an undetectable VL, *below 200 copies/mL*, can be considered uninfectious or U=U.

The actual VL results may vary depending on the sample collected and assay used but any result reported as < 200 copies/mL can be considered undetectable.^7^

*Source*: Authors’ own work. Prevention Access Campaign,^7^ Rodger et al.,^9^ Cohen et al.,^10^ Baeten et al.^19^

In the context of U=U, all other safer conception strategies and approaches discussed in the following represent additional safety measures that should be offered to all couples and may assist those couples who remain anxious and prefer to use additional strategies for their own peace of mind.

However, these strategies do remain critically important options for those couples where viral suppression cannot be confirmed, because it is unavailable as a result of local resource constraints or where maintained VL suppression cannot be assured. They also apply during the period where the HIV-positive partner has not yet started or only recently started ART and is not yet virally suppressed. It is essential that providers remain aware that many HIV-positive people may not be able to attain an undetectable VL because of barriers to treatment access or adherence; others may have ART primary acquired drug resistance or secondary resistance as a result of previous ART exposure, and some may choose not to access HIV testing or, once diagnosed, may not feel ready to start treatment.^7^ In these situations, these other strategies, including PrEP for the HIV-negative partner, provide important options.

### Achieving pregnancy as safely as possible – Choosing appropriate options

**FIGURE 2:**
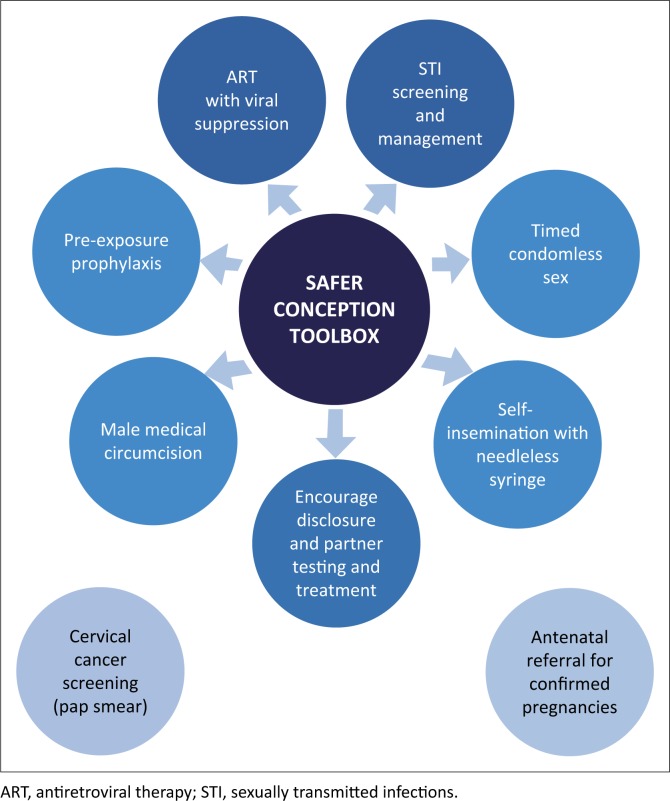
Prepregnancy counselling: Prevention options.

Figure 2 gives an overview of a range of strategies available to HIV-affected individuals or couples seeking to achieve pregnancy. The choice of strategies depends on what is appropriate, based on the HIV status of each partner, clinical considerations, available resources and, most importantly, client preferences (see section ‘Integrated package of care’, Table 3). Table 4, at the end of this chapter following the section ‘Medical male circumcision’, presents the recommended strategies for serodifferent, seroconcordant and sero-unknown couples in more detail.

ART with viral suppression and regular STI screening and management form the foundation of safer conception support. In cases where the partner’s serostatus or VL is unknown, other modalities such as PrEP become critical.

### Antiretroviral therapy, viral suppression and pregnancy planning

For couples who plan to achieve pregnancy through condomless sex, the most important determinant of HIV transmission risk is plasma HIV VL. Effective ART reduces the plasma and genital HIV VL in the infected individual to undetectable levels.^43^ Plasma HIV levels generally correlate positively with the concentration of HIV in genital secretions, rectal mucosa and saliva, although inflammation can stimulate local replication,^44^ *which is why screening for STIs is such an important component of safer conception care*.

Many HIV-affected couples still have much anxiety about trying to conceive in the context of HIV risk. It is important to share with them the robust evidence around U=U in order to provide evidence-based reassurance that they can, in fact, reduce the risk of HIV transmission between partners, and from mother-to-child, to zero if the positive partner or partners are established on ART and confirmed virally suppressed with maintained high levels of adherence over the long-term (see Box 2).^7^

#### Antiretroviral therapy initiation

- As per globally accepted WHO guidelines, all newly diagnosed HIV-positive individuals, and those known to have HIV infection but not yet accessing ART, should be counselled and initiated on ART as soon as possible as part of the UTT approach. The health benefits for the individual of starting ART soon after diagnosis, regardless of CD4+ count, as well as the impact on HIV transmission risk, must be explained to the individual or couple.^45^
- Clients should be counselled about the importance of daily adherence to treatment.
- Antiretroviral therapy initiation should be undertaken as per existing local guidelines, with appropriate screening for underlying opportunistic infections and considering any contraindications to first-line therapy, including renal dysfunction.^13^
- Clients should be reassured that use of ART during pregnancy is considered safe. The benefits of sustained viral suppression far outweigh any potential minimal risks of abnormality in the foetus as a result of teratogenic effects.^18,46^

### Key information and recommendations regarding dolutegravir safety in the periconception period and early pregnancy

At the time of writing (September 2018):

- Preliminary findings from an observational study in Botswana suggest an increased risk of NTDs in infants born to women who conceived while taking dolutegravir.^47^ Previous data concerning dolutegravir exposure in pregnancy, including antiretroviral pregnancy registry data, clinical trials and post-marketing surveillance, had not reported any increased risk of NTDs.
- There is no evidence of safety concerns with dolutegravir use beyond eight weeks of the first trimester. Women can be safely initiated on dolutegravir if they are already pregnant and beyond eight weeks.
- This dolutegravir safety signal highlights the importance of integrating routine fertility intentions screening into HIV care for women of childbearing potential.
- It is imperative that providers be aware of local guidelines relating to dolutegravir use around the time of conception and early pregnancy.
- As further evidence related to the safety of dolutegravir around the time of conception and early pregnancy is likely to emerge in the future, we strongly advise that readers consult the most recent national or international guidance on this specific question.
- This guideline promotes the woman’s right to choose whether to use dolutegravir or efavirenz, based on access to appropriate information about potential dolutegravir safety concerns and how this relates to her own fertility intentions.
- The Southern African HIV Clinicians’ Society (SAHCS) recommends that:
  ■ A woman-centred approach should be adopted: healthcare providers should give women information and options to allow for informed choices about using lifelong ART regimens.
  ■ All women of childbearing potential who are being considered for dolutegravir initiation must be counselled about the possible risks of becoming pregnant while on dolutegravir and should be screened for active fertility intentions.
  ■ All women of childbearing potential who do not have active fertility intentions should be advised about the importance of using a reliable contraceptive method and should be actively linked to the relevant services. These women should also be advised to discuss with their ART provider should their fertility intentions change at any time in the future or if they are planning to discontinue a reliable form of contraceptive.
  ■ Women, including adolescents, of childbearing potential who desire pregnancy or are unable or choose not to access reliable contraception should be counselled about the potential risks and benefits of a dolutegravir- versus efavirenz-based ART regimen and offered the choice. Documentation of this discussion, including consent for those choosing dolutegravir-based ART, is essential.
  ■ Women, including adolescents, on effective contraception or not of childbearing potential may initiate dolutegravir-based first-line ART.
  ■ All pregnant (from eight weeks after conception) and breastfeeding women, including adolescents, may initiate dolutegravir-based first-line ART.
  ■ Any HIV-positive woman of childbearing potential who is already on dolutegravir and plans to become pregnant should be adequately counselled about the potential risks and benefits of dolutegravir- versus efavirenz-based ART and offered the choice of dolutegravir- or efavirenz-based ART. This discussion should be documented, along with consent from those women opting for dolutegravir-based ART.
  ■ Providers should make clinical notes of any discussion, particularly if a woman of childbearing potential chooses to start dolutegravir so that, in the event she does become pregnant on dolutegravir, there is documentation of NTD risk counselling.
  ■ If pregnancy is confirmed in the first eight weeks while a woman is taking dolutegravir, she should be adequately counselled about the potential risks and benefits of dolutegravir- versus efavirenz-based ART and offered the choice of dolutegravir- or efavirenz-based ART. This discussion should be documented, along with consent from those women opting for dolutegravir-based ART. The risk and benefits of switching during pregnancy should also be discussed. Switching is associated with a small risk of viraemia in a previously virologically suppressed patient, which may result in risk of MTCT and resistance.
  ■ For those women opting for efavirenz-based ART, once beyond eight weeks, a woman who does achieve pregnancy may consider switching to dolutegravir in order to benefit from its more robust nature throughout the rest of pregnancy, breastfeeding and lifelong. However, risk and benefits of switching during pregnancy should also be discussed. Switching is associated with a small risk of viraemia in a previously virologically suppressed patient, which may result in risk of MTCT and resistance.
  ■ Women who become pregnant on dolutegravir should be referred for evaluation of birth defects including NTDs, using foetal ultrasound examination around 18–20 weeks gestation. Should a NTD or other congenital abnormality be identified, counselling about the option to terminate the pregnancy should be provided. Continuation or switch of dolutegravir will depend on the gestation at which pregnancy is confirmed. If the woman is already beyond eight weeks in the first trimester, dolutegravir can be safely continued.
  ■ A specialist opinion should be sought for women taking dolutegravir as part of second- or third-line ART regimens, where options to switch may be limited.
  ■ Any adverse outcome observed in a woman of childbearing potential who is on dolutegravir must be actively reported to the provider’s relevant pharmacovigilance or regulatory body so that more data pertaining to dolutegravir safety can be accumulated to inform future recommendations. Where possible, all outcomes, including normal outcomes, should be reported to the relevant body to enable more rapid accumulation of safety data.

#### Recommended duration of antiretroviral therapy before attempting to achieve pregnancy

- Several fertility guidelines recommend that the HIV-positive partner(s) should be on ART for *at least six months* before attempting pregnancy to ensure sustained viral suppression.^18,20,35^ This is particularly important in *settings without access to VL monitoring* as those who start with higher VLs pretreatment may need longer to suppress and there is no way to confirm this.
- It is important to counsel couples from the outset to manage their expectations around timelines and that the advice is, where possible, to wait until the HIV-positive partner has been on ART for six months. Short-term contraception should be offered to individuals or couples during this waiting period and consistent male or female condom use should be emphasised.
- Some couples may find it difficult to wait this long. There are two options in this situation:
  ■ Where VL monitoring is available, the VL may be checked at three months on ART. If viral suppression is confirmed, the couple may begin attempting pregnancy at this point, provided strict treatment adherence is emphasised.
  ■ PrEP may be used as a prevention bridge for serodifferent couples to cover them until the HIV-positive partner has been on ART for at least six months or until VL is undetectable (this is described further in the section ‘Achieving pregnancy as safely as possible – Choosing appropriate options’, Table 2).^19^
- **Clients already established on ART:** If available, the VL should be rechecked to confirm viral suppression before pregnancy attempts are undertaken. If not virally suppressed, treatment should be optimised as per current guidelines and the individual or couple should be encouraged to wait for viral suppression to be confirmed before attempting to achieve pregnancy. Where possible, viral suppression should be confirmed after three months following a switch to any new ART regimen. There should also be evidence of sustained high levels of adherence and immune reconstitution, where necessary, before the couple starts trying for pregnancy. Where the HIV-positive partner is not virally suppressed despite more than six months on ART, PrEP should be offered with caution because of the possibility that accumulating antiretroviral drug resistance may be driving the detectable VL. In this situation PrEP may not be effective as resistant virus may be transmitted to the HIV-negative partner, as has been seen in some case reports of PrEP failure.^48,49,50^ However, as these case reports emphasise, transmitted resistance remains very rare and PrEP has been proven effective for huge numbers of people at risk of HIV infection with very few failures reported globally to date.
- **Viral load monitoring and switching of regimen:** In couples trying to achieve pregnancy, VL monitoring should be conducted at least six-monthly to ensure sustained viral suppression. In resource-intensive settings, this may be increased to three-monthly, in line with current prevention of mother-to-child transmission (PMTCT) guidelines where pregnant and breastfeeding women are monitored every three months:
  ■ Viral load > 1000 copies/mL^3^: advise to wait until viral suppression is (re)established. Explore barriers to adherence and resolve if possible. Discuss the implications of a detectable VL, including possible transmission of drug resistance, and provide support for improved adherence. Many clients will (re)suppress at this point if provided with adequate counselling and adherence support.
  ■ A repeat VL should be taken after one month of supported adherence to confirm (re)suppression. If the second VL remains > 1000 copies/mL, or there has not been at least a one-log drop in the VL value, then the possibility of treatment failure because of drug resistance should be considered, and switching from first-line ART to a recommended second-line option, as per current guidelines, should be undertaken.^13^
  ■ Should a regimen switch be required, the couple should again be advised to wait until suppression of VL is established on the new regimen. Consistent condom use should be encouraged and the risk of transmitting a resistant strain of virus explained to both partners, whether in a serodifferent or seroconcordant relationship, as reinfection with a resistant virus may occur.
  ■ Viral loads between the limit of detectability (assay dependent) and 1000 copies/mL are associated with a very low risk of transmission and hence a couple may be advised that they could continue trying to achieve pregnancy provided they are willing to accept this very low risk. Condomless sex acts should be limited to the periovulatory fertile window to minimise risk exposure. Information about assisting clients to determine their fertile window is provided later in this document (see Appendix 2). The client should be provided with adherence support and his or her VL should be monitored more closely, preferably three-monthly, to ensure that the low-level viraemia is not the beginning of treatment failure with a subsequent rise in VL.^51^ The provider must also ensure comprehensive and regular STI screening as any concurrent, active STI in either partner would increase the risks of HIV transmission in this context.^52^ Serodifferent couples in this situation may also choose to use PrEP as an additional strategy.

**TABLE 2:** Overview of safer conception strategy options for individuals and couples according to HIV dynamic.

Strategy	Serodifferent: known M-/F+	Serodifferent: known M+/F-	Seroconcordant: both known +	Sero-unknown: index + / partner ?	Sero-unknown: index - / partner ?
HIV testing	Recommend repeat testing of male at first visit and at least three-monthly during pregnancy attempts until risk exposure ends.	Recommend repeat testing of female at first visit and at least three-monthly during pregnancy attempts and any resulting pregnancy and breastfeeding.	N/A	Explore HIV disclosure to unknown partner; encourage HIV testing of partner where possible.	Encourage HIV testing where possible of unknown partner. Repeat testing for negative index partner at least three-monthly.
Appropriate ART with confirmed viral suppression or at least six months treatment with full adherence	✔	✔	✔	✔	✔ For unknown partner if engaged and tests HIV-positive
STI screening and management†	✔	✔	✔	✔	✔
PrEP	Recommended if HIV-positive partner not confirmed VL < 200 copies/mL *or* not on ART ≥ 6 months with optimal adherence *or* client preference	Recommended if HIV-positive partner not confirmed VL < 200 copies/mL *or* not on ART ≥ 6 months with optimal adherence *or* client preference	N/A	If unknown partner engages in testing and confirmed HIV-negative, offer as per known serodifferent couples	Recommended if partner remains untested or is newly diagnosed and needs to be established on ART
MMC	Recommended for all HIV-negative males; discuss the potential benefits for HIV-positive males				
Timed condomless sex	Recommended if positive partner(s) not confirmed VL < 200 copies/mL or viral load monitoring unavailable or client preference			May not be practical but can discuss	
Self-insemination with needleless syringe	✔	N/A	N/A	N/A	Discuss if male index. May not be practical.
Disclosure or partner engagement support‡	✔	✔	✔	✔	✔
Cervical cancer screening (as per local guidelines)	✔	✔	✔	✔	✔
Early linkage to ANC care if pregnancy confirmed	✔	✔	✔	✔	✔

ART, antiretroviral therapy; PrEP, pre-exposure prophylaxis; VL, viral load; MMC, medical male circumcision; ANC, antenatal care; STI, sexually transmitted disease; N/A, not applicable.

Note: All strategies that are relevant to each individual or couple should be offered and discussed so that the client(s) can make an informed choice of which strategy, or combination of strategies, they would prefer to use during pregnancy attempts.

† , Includes all male and female clients who access service; where possible, contact or trace absent partner if index partner screens positive for any STI.

‡ , All clients who have not disclosed should be supported to safely do so. All clients attending alone should be encouraged to come with their partners, but disclosure and partner attendance are not requirements for safer conception service provision.

### Antiretroviral drugs in pregnancy

We recommend standard first-line, second-line and third-line regimens be used in pregnancy.^6^ Based on the accumulated evidence, we endorse the WHO guidance that efavirenz can safely be used in pregnancy and in women who intend to become pregnant.^14^ Their guidance was based on a meta-analysis that found that the incidence of NTDs and all congenital abnormalities among women exposed to efavirenz in the first trimester was similar to that of the general population.^15^ The FDA category D classification of efavirenz, and accompanying package insert, should be discussed with women, explaining that this was based on animal studies; human cohort studies have not demonstrated an increased risk of congenital abnormalities, but there is a background low risk of congenital abnormalities in all pregnancies, unrelated to drugs.^53^ Providers are also referred to the section ‘Key information and recommendations regarding dolutegravir safety in the periconception period and early pregnancy’ which presents recommendations pertaining to dolutegravir use in women of childbearing potential.

Providers are encouraged to maintain high levels of pharmacovigilance, reporting any adverse outcomes seen in women on any ART drug during pregnancy (for either treatment or prevention) to their local ART pregnancy registry.

### HIV-positive women not yet on antiretroviral therapy and confirmed pregnant

Many countries, including South Africa, have implemented UTT ART guidelines so all people testing positive for HIV should be offered immediate ART, regardless of their CD4+ count or clinical staging. Prior to this, South Africa had implemented the Option B+ PMTCT strategy, which made provision for any women commencing ART during pregnancy to then continue ART lifelong.^13^ Any HIV-positive woman presenting pregnant before HIV diagnosis or ART initiation should be initiated as soon as possible, preferably on the day that pregnancy is confirmed, and then reviewed in one week to follow up on baseline bloods and continue with treatment literacy and adherence counselling. Refer for antenatal care (ANC)/PMTCT services as soon as pregnancy is confirmed or as early as possible in pregnancy.

Similar to horizontal transmission, viral suppression is critical to reduce the likelihood of vertical HIV transmission to the foetus *in utero* or to the infant during delivery. The longer a woman is on ART during pregnancy, the better the outcomes – for herself and for her infant – because of an increased likelihood of an undetectable VL at delivery, thus preventing peripartum MTCT.^54,55^ Undetectable VL also reduces the risk of HIV transmission during breastfeeding as well as transmission to any serodifferent partner during this time.

### Pre-exposure prophylaxis

#### Pre-exposure prophylaxis for serodifferent and sero-unknown couples

It is recognised that for many women, the involvement of their male partners in couples counselling and testing or in any part of the health system remains a challenge.^56,57,58^ While health systems grapple with the challenge of developing male-attractive health services, it is important that HIV-seronegative women and their unborn offspring be protected even when there are demands for condomless sex.

There is now considerable evidence that PrEP, in the form of tenofovir and emtricitabine, effectively prevents HIV transmission in serodifferent couples.^59,60^ Studies have also shown efficacy with tenofovir alone, but to date WHO recommendations are for the combination of tenofovir and emtricitabine.

PrEP may thus offer additional risk reduction for the HIV-negative partner in a serodifferent relationship or be the only protection option in some cases. However, evidence and careful modelling have shown that PrEP would be unlikely to provide additional risk reduction benefit where the HIV-positive partner has been on ART for at least six months with optimal adherence or has confirmed viral suppression < 200 copies/mL by formal laboratory or point of care monitoring. In this situation, the risk of HIV transmission is already considered to be so low that PrEP would not add a further benefit.^61^

However, PrEP is advisable in the following circumstances:

- while the HIV-positive partner is becoming established on ART, referred to as a ‘bridge’^19^
- where the HIV-positive partner does not want to engage in care or refuses ART
- where the HIV-negative partner has concerns about the reliability of their HIV-positive partner’s adherence to treatment
- where VL monitoring is not available and there are concerns that the HIV-positive partner may not be virally suppressed, for example if there are known to be occasional interruptions to drug supply that are beyond the individual’s control
- if the HIV-negative partner wishes for additional protection despite confirmed viral suppression and is concerned about condomless sex timed to the fertile window
- where the HIV-negative partner is unable to ascertain their partner’s HIV status and so may be exposed to unknown HIV risks if embarking on condomless sex
- in any situation where the HIV-negative partner in a serodifferent or sero-unknown relationship chooses, after counselling, to use PrEP because of personal preference.

#### Using pre-exposure prophylaxis for HIV prevention

- HIV-negative individuals opting to use PrEP should be managed according to existing PrEP guidelines.^59^
- Important points include the following:
  ■ Confirmation of HIV-negative status and screening for signs or symptoms of acute HIV seroconversion before PrEP initiation.
  ■ Screening for underlying renal dysfunction and hepatitis B infection.
  ■ Counselling about current recommendations on daily use of PrEP to ensure effectiveness.
  ■ The pharmacokinetic modelling data that have been used to estimate the dosing period required before protection varies from seven to 21 days. This should be discussed with clients and, where possible, given the non-urgent situation, it would be advisable to recommend initiating PrEP at least 20 days prior to trying to achieve pregnancy.^59^ Therefore, a lead in time of a minimum of 20 days is recommended for females and for male partners in heterosexual relationships who will be undertaking condomless vaginal-penile sex. No data currently exist concerning time to attain effective tissue levels in men who do not have sex with men.^62,63,64^
  ■ Pre-exposure prophylaxis should be continued daily while trying to achieve pregnancy and for one month after returning to consistent condom use to cover a 28-day period since the last known HIV risk exposure.
  ■ Repeat HIV testing should be conducted at least three-monthly during PrEP use.

#### Pre-exposure prophylaxis and pregnancy

Where pregnancy is confirmed in a woman taking PrEP, the risks and benefits of continuing PrEP should be carefully discussed. There is currently no clear evidence of harmful effects resulting from significant *in utero* foetal exposure to tenofovir or emtricitabine. A systematic review that included studies observing HIV-negative and hepatitis B-positive women who took tenofovir and emtricitabine treatment throughout their pregnancies provided reassuring evidence including the following:^65^

- no difference in low birth weights between tenofovir and control regimens
- no increase in reported birth defects (for both HIV-infected and not infected women)
- no significant difference in infant growth
- no significant impact on maternal health.

However, *increased neonatal mortality risk with tenofovir exposure* has been noted in two studies in relation to significantly higher very preterm delivery (< 34 weeks) and associated neonatal mortality when women were taking a tenofovir-based ART regimen compared to non-tenofovir ART regimen.^65,66^ There also remains a need to assess longer-term infant growth and bone effects.

A limited number of PrEP studies including HIV-negative women who became, or were already, pregnant when taking PrEP are starting to emerge with similarly reassuring data to that seen among women using tenofovir or tenofovir and emtricitabine (Truvada^®^) for HIV or hepatitis B treatment.^18,67,68,69,70^

Based on the accumulating evidence, the risks for pregnant or breastfeeding HIV-negative women using PrEP for HIV prevention are thought to be minimal and are outweighed by the prevention benefit where the woman remains at substantial risk of acquiring HIV during this time.^71^ It is important to explain to women who are taking PrEP and are confirmed pregnant that most current evidence is based on women using tenofovir or tenofovir and emtricitabine (Truvada^®^) as a form of treatment for an existing infection (HIV or hepatitis B), not as a prevention strategy. The woman’s risk of HIV acquisition during pregnancy and breastfeeding should be carefully considered and, where a woman remains at substantial risk of HIV infection, SAHCS, in alignment with recent WHO recommendations, advises that the woman be maintained on PrEP throughout pregnancy and during breastfeeding. This is particularly important for women who cannot negotiate consistent condom use and where the partner is either of unknown HIV status in a high HIV prevalence setting or known to be HIV-positive but not accessing care, not virologically suppressed or not adhering optimally to treatment. The benefits of continuing PrEP throughout pregnancy and breastfeeding in order to avoid seroconversion in the pregnant or breastfeeding woman, with consequent significant risk of MTCT, are considered to far outweigh any potential risk of tenofovir or emtricitabine exposure to the foetus or breastfed infant.^59,60,65^

Note for South African providers: Currently the National South African Department of Health (DoH) Guidelines do not recommend PrEP during pregnancy because of regulatory issues as the Medicines Control Council regulatory approval includes a paragraph stating that PrEP use is contraindicated in pregnancy. Studies are currently underway to increase the exposure of pregnant and breastfeeding women to tenofovir-based PrEP and it is hoped that the DoH guidelines will soon be updated to synchronise with WHO recommendations. In this context, the final decision should be made through full consultation between clinician and client with a balanced consideration of risks and benefits.

Oral tenofovir-based PrEP is also considered safe in breastfeeding because:

- Transfer of tenofovir from maternal plasma to breast milk is limited.
- Infant exposure was found to be > 200 times lower than the proposed infant therapeutic dose.
- Tenofovir was not detected in 94% of infant plasma samples.^71,72^

**Note:** Pre-exposure prophylaxis use in pregnancy should be accompanied by high levels of pharmacovigilance. Providers are strongly encouraged to report any adverse maternal or infant outcomes to their national regulatory authority and any established PrEP or ART safety registry or committee.

### The importance of HIV retesting

- All HIV-negative partners, whether choosing to take PrEP or not, should be retested regularly for HIV. It is recommended that this be done at least every three months during pregnancy attempts.
- HIV acquisition during pregnancy, and immediately following pregnancy, remains high despite increased access to, and initiation of, ART in the general population.
- In South Africa, the maternal HIV incidence rate was 10.7 per 100 person years (PY) and 12.4 per 100 PY in urban health facilities in 2013.^73,74^
- In a recent meta-analysis, MTCT risk was significantly higher among women with newly acquired HIV infection during pregnancy or breastfeeding compared to those already known to be HIV-infected in the post-partum period (odds ratio [OR] 2.9, 95% confidence interval [CI] 2.2–3.9) or in the pregnancy and post-partum periods combined (OR 2.3, 95% CI 1.2–4.4).^75^
- For females who achieve pregnancy, retesting should be continued at least three-monthly throughout pregnancy and breastfeeding, along with counselling about the continued use of risk reduction strategies to avoid seroconversion during pregnancy or the breastfeeding period, as this is associated with higher health risks to women (higher maternal morbidity and mortality) and very high risks of MTCT because of the high VLs seen during acute HIV infection.^75^
- HIV-negative partners who have proven seroconversion at retesting should be initiated on ART, as per the UTT approach.
- As HIV self-screening becomes more widely available and acceptable,^76^ this provides an important alternative for HIV-negative partners who may not want to attend a clinic to access regular testing. Education should be provided about reliable, quality assured self-screening brands as they become available in each country, as well as advice about how regularly to test and what to do should a self-screening result be positive. Any positive self-screening result must be confirmed according to standard algorithms for facility-based or community-based testing.^77^

### Post-exposure prophylaxis

Post-exposure prophylaxis (PEP) is recommended for accidental exposure to HIV, either occupational or non-occupational.^78^ It is, however, no longer recommended as a key strategy for persons trying to achieve pregnancy because of the repeated risk exposures usually required to achieve pregnancy, which then necessitates repeated rounds of PEP. Any individual trying to achieve pregnancy who presents for PEP after condomless sex within the last 72 hours should be provided with a single course of PEP as per existing guidelines. They should then be counselled and offered to transition to daily PrEP, if available, as a preferred prevention method because of the expectation of repeated HIV risk exposures as they continue to try and achieve pregnancy.

### Other components of prepregnancy workup

Table 3 (see section ‘Integrated package of care’) shows recommended basic investigations that may be undertaken in primary care facilities as part of prepregnancy workup in both resource-limited and resource-intensive settings.

**TABLE 3:** Prepregnancy screening for HIV-positive individuals desiring a child in resource-limited and resource-intensive settings.

Female partner	Male partner
Viral load on ART CD4+ count Syphilis and hepatitis B serology Syndromic screening for other STIs or †hepatitis A, B and C serology, syphilis, gonorrhoea, chlamydia, trichomonas, HSV-2 screening Haemoglobin or †full blood count Visual inspection of the cervix and pap smear/VIA in absence of documented normal result in the previous 12 months and/or †HPV screening Non-communicable diseases screen (hypertension, diabetes, weight review, †cholesterol) †CMV, rubella, toxoplasmosis †If infertility suspected or no pregnancy after six months, refer for fertility assessment	Viral load on ART CD4+ count Syphilis and hepatitis B serology Syndromic screening for other STIs or †hepatitis A, B and C, syphilis, gonorrhoea, chlamydia, trichomonas, HSV-2 screening Non-communicable diseases screen (hypertension, diabetes, weight review, †cholesterol) †If difficulty conceiving: refer for semen analysis and fertility assessment

VIA, visual inspection with acetic acid; CMV, cytomegalovirus; HSV, herpes simplex virus; ART, antiretroviral therapy; STIs, sexually transmitted infections; HPV, human papilloma virus.

Note: CD4+ count is not required for ART eligibility but rather to assess HIV advancement and immunological well-being.

† , Screening that is typically only available in resource-intensive settings.

#### Integrated package of care

Integrated care should be strongly supported. Clients should be counselled prior to their prepregnancy workup about these investigations and why they are important for their health.

These investigations include the following:

- HIV-related investigations.
- Syphilis and hepatitis B screening, haemoglobin measurement and physical examination including a full genital examination to identify any signs of STIs.
- A Papanicolau (Pap) smear should be performed for all women who do not have a documented normal result within the recommended screening period, based on national cervical cancer screening guidelines. Abnormal pathology, including high grade squamous intraepithelial lesions, should be managed according to national guidelines^79^ prior to trying to achieve pregnancy, and the couple should be advised to defer pregnancy attempts until the abnormal result has been managed further and any healing following excisional biopsy has taken place (which usually takes six weeks).
- Should a woman require extensive excision, including cone biopsy, there may be a slightly increased risk of pregnancy loss, preterm delivery and preterm premature rupture of membranes, and the client should be counselled about this.^80^ Good communication should be maintained between the cervical cancer screening and gynaecology team and the safer conception provider to ensure appropriate follow-up should pregnancy be achieved. National guidelines should be consulted to inform referral of women with abnormal pathology to the appropriate specialised services.
- Basic investigations may be extended, where resources allow, to include a full screen for TORCH infections (toxoplasmosis, rubella, CMV, herpes simplex and other congenital infections), along with a full hepatitis screen and full blood count.

### Sexually transmitted disease screening and treatment

All males and females should be screened, using a basic questionnaire^81^ for new STI symptoms at each visit. If either partner is confirmed, or suspected, to have an STI then both partners should be managed according to local guidelines. The treatment course must be completed before pregnancy attempts begin. In most settings, STI screening and management will be based on the syndromic approach.^81^ However, because of the high rate of asymptomatic STIs seen in many populations,^82^ if available, tests for *Chlamydia trachomatis*, gonorrhoea (*Neisseria gonorrhoeae*), *Trichomonas vaginalis*, syphilis and herpes simplex virus-2 should be performed and any positive results managed by treating the client and his or her partner according to current treatment algorithms.

Effective STI screening is not only important for HIV transmission risk reduction but also to avoid the negative consequences of STI infections during pregnancy. These include higher risks of miscarriage, intrauterine growth retardation, premature labour and delivery, and the possibility of neonatal, congenital STI infection.^83^

### Timed condomless sex

- Timed condomless sex is an additional safer conception strategy that may be used by some couples.^35^ It is recommended for all HIV-affected couples where an undetectable VL cannot be confirmed.
- The risk of STI transmission or acquisition when condoms are not used should always be noted.
- In those couples where U=U applies – one or both partners are HIV-positive with a sustained undetectable VL and maintained adherence – careful timing of condomless sex to the woman’s peak fertile days is no longer considered necessary. This is because correctly identifying the days of peak fertility can be difficult and restricting couples to certain days may not actually increase their chances of conceiving and may instead create confusion and/or stress within the relationship by disrupting their usual patterns of intercourse. In presumed fertile, U=U couples, it may be best to avoid such ‘meddlesome’ activity^84^ and leave the couple to have regular condomless sex without strict timing.
  ■ Although viral shedding in semen has been reported to occur even in men who are fully suppressed on ART,^85^ recent evidence indicates that this detectable virus in seminal fluid may be particles of HIV RNA or DNA rather than entire, viable virions, which are required to be infectious; therefore, such detectable virus should not lead to HIV transmission.^7^
- Because some HIV-affected but U=U couples may prefer to reduce risk as much as possible, the pros and cons of timed coitus should be discussed with all couples.
- For seroconcordant and serodifferent couples where viral suppression is not possible, or cannot be confirmed, and for individuals with a partner of unknown HIV status, the option of having condomless sex timed to the window of ovulation in order to limit HIV risk exposure while trying to achieve pregnancy should be discussed. For most couples this is the most feasible risk reduction strategy available to them. Partners must be aware of the potential risk of HIV transmission or reinfection and should understand that this risk increases on a continuum – the higher the VL, the higher the risk of HIV transmission per condomless sex act.^86^ Couples should be offered information on how to time condomless intercourse to the peak fertile window (see Appendix 2).
- Couples should be informed that timed condomless sex should be combined with other risk reduction strategies, including ART adherence and, where appropriate and available, PrEP (see section ‘Medical male circumcision’, Table 4).
- For HIV-positive seroconcordant couples, the possibility of reinfection, or transmission of different or resistant viral strains, should be discussed, particularly if one partner is not virally suppressed and there is a concern about treatment failure and possible ART drug resistance. This may become more common as the HIV epidemic evolves and more individuals develop resistance to first-line therapy.

**TABLE 4:** Summary of optimal risk reduction strategies for resource-limited and resource-intensive settings, according to the HIV status of the couple.

Partner	Seroconcordant (female and male HIV-positive)	Serodifferent (male HIV-positive, female HIV-negative)	Serodifferent (female HIV-positive, male HIV-negative)	Sero-unknown (one partner has unknown HIV status)
Female partner	ART recommended for six months before attempting pregnancy. Encourage high levels of adherence. CD4+ monitoring as per guidelines. Recommend CD4+ > 200 cells/mL prior to pregnancy attempts. If available, confirm undetectable viral load and monitor at least six-monthly. Timed condomless sexual intercourse where undetectable viral load unconfirmed or couple chooses as adjunct.	Repeat HIV testing before pregnancy attempts begin, throughout trying period and throughout pregnancy and breastfeeding. Begin trying once male viral load confirmed undetectable. Timed condomless sexual intercourse during fertile period where undetectable viral load unconfirmed or couple chooses as adjunct. Consider PrEP, particularly if male partner newly on ART (< six months), not engaged in ART care, adherence concerns or heightened anxiety or where viral load monitoring unavailable. Consider sperm washing and intrauterine insemination.†,‡	ART recommended for six months before attempting pregnancy. Encourage high levels of adherence. CD4+ monitoring as per national guidelines, recommended CD4+ > 200 cells/mL prior to pregnancy attempts. If available, confirm undetectable viral load† and monitor at least six-monthly while attempting conception. Where undetectable viral load unconfirmed, or as an adjunct measure chosen by couples, timed condomless sex limited to peak fertile window *or* intravaginal self-insemination using condoms without spermicide *or* sperm collection with intrauterine insemination.†	**HIV-negative female with unknown male status:** Repeat HIV testing throughout exposure period. Offer PrEP. Provide support to try and engage male partner. Timed condomless sexual intercourse. **HIV-positive female with unknown male status:** ART recommended for six months before attempting pregnancy. Encourage high levels of adherence. CD4+ monitoring as per national guidelines and recommend CD4 > 200 cells/mL prior to pregnancy attempts. If available, confirm undetectable viral load† prior to beginning pregnancy attempts and monitor at least six-monthly while trying. Provide support to try and engage male partner. Timed condomless sexual intercourse once female viral suppression confirmed as male partner status unknown.
Male partner	ART recommended for six months before attempting pregnancy. Encourage high levels of adherence. CD4+ monitoring as per national guidelines. If available, confirm undetectable viral load and monitor at least six-monthly while attempting pregnancy.	ART recommended for six months before attempting pregnancy. Encourage high levels of adherence. CD4+ monitoring as per national guidelines. If available, confirm undetectable viral load† before pregnancy attempts begin and monitor at least six-monthly while trying. May opt for sperm assessment and sperm washing with HIV PCR.† Medical male circumcision may be offered.	Repeat HIV testing before pregnancy attempts begin, throughout trying and at end of window period from last exposure. Recommend male medical circumcision. Consider PrEP where female newly established on ART (< six months), not accessing treatment, no viral load monitoring available, adherence concerns or high levels of anxiety regarding HIV acquisition.	**HIV-negative male with unknown female status:** Repeat HIV testing throughout. Offer PrEP. Provide support to try and engage female partner. Timed condomless sexual intercourse. Encourage medical male circumcision. **HIV-positive male with unknown female status:** ART recommended for six months before attempting pregnancy. Encourage high levels of adherence. CD4+ monitoring as per national guidelines. If available, confirm undetectable viral load† prior to pregnancy attempts and monitor at least six-monthly throughout pregnancy attempts. Provide support to try and engage female partner. Timed condomless sexual intercourse once male viral suppression confirmed as female status unknown.

PrEP, pre-exposure prophylaxis; ART, antiretroviral therapy; PCR, polymerase chain reaction.

Note: HIV testing and retesting to be conducted as per national guidelines and with high level quality assurance of testing processes. **All female partners, regardless of HIV status, should be started on folic acid supplementation (per local guidelines) prior to undertaking pregnancy attempts**.

† , Options that are more commonly available in resource-intensive settings, although they may be available in resource-limited settings as well.

‡ , Costly, may not improve HIV prevention, reduced conception rates per cycle compared with natural conception in couples where no fertility issues are identified.

### Self-insemination

- For male HIV-negative serodifferent couples an alternative to timed, condomless sex is intravaginal self-insemination.^87^ This technique is low cost, requiring only a clean, needleless syringe and a condom without spermicide or a clean specimen cup (see Appendix 3).
- The healthcare provider can teach the couple how to do this procedure in their own home:
  ■ Couples should have timed sex using a condom (without spermicide), aspirate the semen from the condom using a needleless syringe and then insert the syringe into the vagina and slowly ‘inject’ the semen.
- The man can also masturbate and ejaculate into a clean specimen bottle from which the semen can be drawn up into the syringe and inseminated into the female’s vagina within the hour, being kept at body temperature if there is any delay between ejaculation and insemination. Reassure couples that any child conceived will still be genetically the man’s child, as this has been raised as a concern in some qualitative studies exploring community perceptions of this option.^38^
- Alternatively, if the couple prefers, freshly collected semen can be brought to the clinic and vaginal insemination can easily be performed by the healthcare provider. The semen should be kept at body temperature in a clean specimen jar (not condom) and the couple should be helped at the clinic within the hour.
- Other HIV-affected couples may choose this technique if there is anxiety about HIV transmission when trying to conceive using condomless sex. However, for these couples, there is no additional HIV risk reduction benefit gained from using this technique. It may be an option for couples who experience sexual dysfunction arising because of anxiety about having condomless sex.

### Sperm washing

In resource-intensive settings, a serodifferent couple with an HIV-positive male partner may opt to use sperm washing as a risk reduction strategy.^88^ Following sperm washing, the couple would need to undergo IUI, IVF or ICSI, which are costly and invasive procedures when compared to natural conception. Again, the pros and cons to this approach should be discussed with the couple. As the risk of timed condomless sex is now considered to be so low in the setting of viral suppression with ART, even couples in resource-intensive contexts may prefer to opt for natural conception and avoid the costs, hospital visits and stress that can be associated with utilising IUI or other assisted reproductive technologies.

### Medical male circumcision

HIV-negative men should be counselled on the benefits of male circumcision as an additional HIV risk reduction strategy.^89^ There is also possibly a smaller benefit for partners of HIV-positive men in serodifferent relationships as circumcision may slightly reduce male-to-female HIV transmission risk.^90^ Medical male circumcision (MMC) also reduces the risks of some other STIs and cervical cancer in female partners.^90^ If medical male circumcision is performed, the couple should wait before trying to achieve pregnancy for at least six to eight weeks or longer if there is still discomfort to ensure complete healing of the circumcision wound.

**Note:** Across all groups, male and female partners (where engaged) should be screened regularly for STIs as per existing syndromic screening and management guidelines (or as per available tests and protocols). If a new STI is detected in either partner, pause pregnancy attempts until both partners have completed treatment.

## Clients with no immediate plans for a child

Couples and individuals who wish to prevent a pregnancy in the near future should be offered a reliable contraceptive method of their choice, within a rights-based framework, as per existing national^2,91^ or global^22^ contraceptive guidelines.

Although fertility may be reduced, particularly in advanced HIV disease, the overall incidence of pregnancy is seen to increase following access to ART.^92^ Importantly, the rate of unintended pregnancy remains high among HIV-positive women, just as seen in the general population.^93^

Access to contraception contributes to the goals of EMTCT by strengthening Pillar 2 of the PMTCT programme (prevention of unplanned pregnancies), which remains one of the weakest areas of PMTCT programme implementation in many countries.

Using contraception is also an option for couples who do wish to achieve pregnancy but where certain aspects of their health need to be optimised before pregnancy attempts are undertaken. Initiating short-term contraception in this situation may help to prevent pregnancy being achieved under suboptimal conditions and different contraception options should be discussed. Examples where this may be relevant include when the HIV-positive partner is taking treatment for an acute opportunistic infection such as tuberculosis or other infection such as bacterial STI; where the female’s CD4+ count is still low but expected to improve; where the positive partner is only just initiating on ART and is not yet virally suppressed or where a new non-communicable disease is diagnosed and treatment is being optimised. An important consideration is to use a method with immediate return to fertility, as summarised in Box 3 (‘Special considerations when providing contraception to HIV-positive women’ section). These couples should be encouraged to continue using male or female condoms as part of dual protection.

BOX 3 Expected return to fertility with different contraceptive methods.

If there are no concerns about underlying fertility issues:

- Hormonal contraceptive pills including combined oral contraceptive pill (COCP), progesterone only pill and emergency contraceptive pill, contraceptive implants and IUCDs are all associated with immediate return to fertility after discontinuation or removal.
- NET-EN: three to six months from date of last injection.
- DMPA: six to nine months from date of last injection.
- Male and female sterilisation: permanent, very low success rate with reversal.

Notes:

- Up to 50% of women who discontinue the COCP are pregnant within three months.
- Following discontinuation of a hormonal injectable contraceptive, ovulation and pregnancy can occur before the normal menstrual cycle has been established.
- Fertility return varies depending on how long it takes a woman to fully metabolise the DMPA from her last injection. Women differ in how they metabolise DMPA so there is considerable variability in how long it takes for fertility to return after discontinuation.^100^
- Return to fertility should be clearly explained – it means that the menstrual cycle and ovulation return, but one’s ability to get pregnant depends on many other factors.

*Source*: Author’s own work. Adapted from World Health Organization^22^ and FHI360.^100^

DMPA, depot medroxyprogesterone acetate; NET-EN, norethisterone enanthate; IUCDs, intrauterine contraceptive devices.

### Important points to note when providing contraception services

- Method selection will be based on medical eligibility,^94^ screening and information about contraceptive choices.
- Clients should be encouraged to discuss any new plans to achieve pregnancy with their provider, so that contraception can be discontinued in conjunction with supportive interventions to ensure a safe, healthy pregnancy.
- Informed decision-making is key. Information about mechanisms of action, possible side effects and return to fertility after discontinuing a method should be provided. Myths and misconceptions should be addressed, particularly those relating to contraceptive use as a cause of infertility.
- Partner involvement should be discussed and supported if the woman requests; however, she may choose to make her decision independently of her partner.
- After a woman has given birth, a reliable method of post-partum contraception should be prioritised, with informed consent, to ensure that the next child, if desired, can be planned with appropriate spacing.
- All women should be encouraged to use dual methods, combining their contraceptive choice with the consistent use of male or female condoms to reduce the risk of HIV and STIs. Avoiding STIs, via consistent condom use, is an important consideration for women who may wish to have a child later, as STIs such as *C. trachomatis* are associated with both reduced fertility and risk of miscarriage and pregnancy complications, including intrauterine growth restriction, and premature labour and delivery.^95^

Where an individual or couple communicates that they prefer not to use condoms, the following counselling messages should be covered:^59^

- Where the HIV-positive partner is virally suppressed and/or the HIV-negative partner is on PrEP, not using a condom still has risks:
  ■ Undetectable VLs are dependent on ART adherence and need to be monitored.
  ■ Undetectable VL and/or PrEP prevents HIV transmission but does not prevent other STIs and pregnancy (unless reliable contraception is used).
- A regular STI screening and management plan should be confirmed with the client.
- Vaccination against all vaccine-preventable STIs, for example, hepatitis A and B and HPV, should be offered where possible.
- Information about emergency contraception should be provided.
- Information and counselling should be provided about rights and options in terms of safe abortion should an unintended pregnancy occur.

### Special considerations when providing contraception to HIV-positive women

A range of detailed resources are available that provide guidance on contraceptive provision, including for women living with HIV.^22,94^ Key points are highlighted as follows:

- Numerous effective contraceptive options are available for safe use by women living with HIV. These include barrier methods (male and female condoms), hormonal injectables such as depot medroxyprogesterone acetate (DMPA) or norethisterone enanthate (NET-EN), combined oral contraceptive pills (COCP), subdermal contraceptive implants, intrauterine contraceptive device (IUCD) and levonorgestrel intrauterine system (LNG-IUS).^2,91,94^
- Long-acting reversible contraceptive options, particularly the IUCD, should be encouraged where possible, especially among young women, because of fewer side effects, better adherence and immediate return to fertility following removal. The IUCD is also a recommended option for women who have completed their families but do not wish to access permanent sterilisation.
- Drug interactions, as per the WHO medical eligibility criteria (MEC) and country guidelines, need to be considered, for example:
  1. The efficacy of subdermal implants in preventing pregnancy may be compromised because of drug interactions with certain antiretroviral drugs, such as efavirenz,^96^ and certain other enzyme-inducing tuberculosis (TB) and antiepileptic drugs.^97^ Women who choose this as a method need to be counselled about the risks and the need for consistent male or female condom use as additional protection. However, there is no need to counsel against implant use or advise early removal of implants in women living with HIV.
  2. Non-nucleoside reverse transcriptase inhibitors, particularly efavirenz, interact with the COCP such that there is a marginally higher rate of pregnancies seen in women on efavirenz and COCP, but the pregnancy rate observed was still considerably lower than among women on no form of modern contraception.^96^ Women on protease inhibitors did not have a significantly higher rate of pregnancy.^96,98^ The COCP should, therefore, remain an option for women on these ARVs and is preferable to being on no reliable contraception when the COCP is the woman’s preferred choice. However, as with the subdermal implant, more extensive interactions are seen with TB drugs, particularly rifampicin, as well as antiepileptic medications, so these interactions should be reviewed before proceeding with a final choice for women on one or more of these medications. Again, dual prevention with consistent male or female condom use should be recommended to all women.
- There are various options for women and couples who do not want any further children in the future. These include long-acting reversible methods and voluntary male or female sterilisation. The latter should only be performed after thorough, non-coercive counselling to ensure there is full understanding that this option is permanent and not reversible and in compliance with national guidelines on voluntary male and female sterilisation.

It is also important to note that for HIV-negative women there are concerns that DMPA/NET-EN hormonal injectable contraceptives may be associated with an increased risk of HIV acquisition and transmission.^99^ Any HIV-negative female who chooses this contraceptive option should be counselled about the importance of consistent condom use to prevent HIV-infection, the option of treatment as prevention where her partner is on ART and virally suppressed and the increasing availability of PrEP.

**FIGURE 3:**
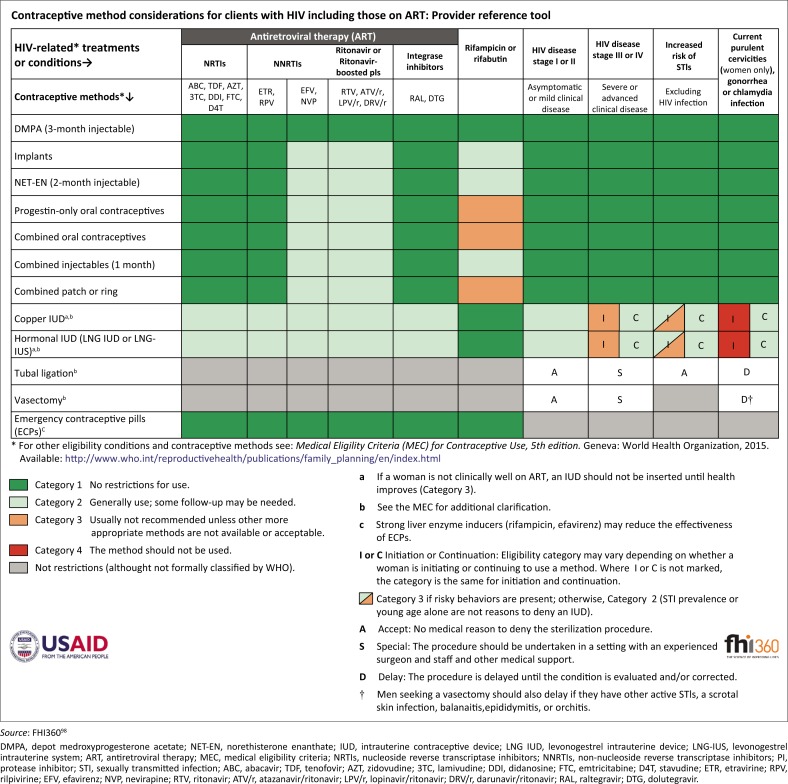
Contraceptive methods for HIV+ individuals, including those on antiretroviral therapy.

## Additional considerations for optimising prepregnancy health

### Identifying and managing comorbidities

- Both partners should be screened for any other comorbidities.
- It is strongly recommended that HIV-positive women should have a CD4+ count of 200 cells/mL or more before considering pregnancy so that their risk of opportunistic infections and deteriorating health during the pregnancy can be minimised. However, many women with a CD4+ count lower than this have successfully carried a healthy pregnancy to term and, for some women, waiting until their CD4+ count has risen to over 200 cells/mL following ART initiation may not be possible, including if they have a slow response or are already over 35 years of age. In this situation the woman should be counselled about the risks of being pregnant with a lower CD4+ count and appropriate prophylaxis should be considered, including cotrimoxazole and isoniazid prophylactic therapy to prevent TB.
- HIV-positive partners should also be screened for opportunistic infections, in particular TB. Should an opportunistic infection be detected that requires a finite period of treatment, the couple should be encouraged to defer pregnancy attempts until the treatment course is complete and the individual has returned to full health.
- HIV-positive women who are on fluconazole because of a history of cryptococcal meningitis or positive cryptococcal antigen should be alerted to the risk of craniofacial and skeletal deformities following first trimester exposure to high-dose fluconazole.^101^ Clients in this situation should be encouraged to defer pregnancy attempts until CD4+ reconstitution has been confirmed to be at least between 100 cells/mL and 200 cells/mL on two separate occasions so that the fluconazole prophylaxis can be safely discontinued without risk of cryptococcal disease relapse.^102^ In the rare situation where CD4+ reconstitution above 100–200 cells/mL does not occur, the risks of continuing versus stopping fluconazole during the first trimester, if pregnancy is achieved, should be discussed.
- Screening should also be offered, where available, for chronic co-infections such as hepatitis B. Where hepatitis B infection is confirmed, this should be managed according to guidelines.^103^ If the couple is confirmed hepatitis B serodifferent then the hepatitis B-negative individual should be offered immunisation before pregnancy attempts are undertaken; the hepatitis B-positive individual should be established on effective therapy and, where possible, the hepatitis B VL should be confirmed to be undetectable. Where the hepatitis B-positive individual is co-infected with HIV, the use of tenofovir and emtricitabine or lamivudine should prove sufficient for hepatitis B infection management as well. A female client with hepatitis B should have her hepatitis B VL monitored during pregnancy and the labour and delivery team should be alerted to the possibility of congenital hepatitis so that the newborn can be provided with immunoglobulin and immunisation, where available, as per current recommendations.^103^ Where there is no evidence of hepatitis B infection or immunity (surface antigen and surface antibody negative) in either partner then vaccination should be considered, particularly in higher prevalence settings.
- Female clients should be screened for non-communicable diseases including hypertension and diabetes mellitus. Where present, blood pressure and/or glucose control should be optimised prior to pregnancy attempts being undertaken. Complex cases should be referred to the relevant specialist for assistance with management optimisation. Where feasible, acknowledging resource constraints, male clients should also be screened and managed for non-communicable diseases.
- Obesity is increasing globally. Should one or both partners be obese then possible ways to assist with appropriate weight reduction should be discussed. Obesity is associated with reduced fertility in both men^104^ and women,^105^ and this may be an important motivational factor to encourage weight loss. Obese clients, especially pregnant women, should be screened regularly for hypertension and diabetes mellitus.
- For all other chronic conditions that require treatment throughout pregnancy, it is important to review all medications for potential teratogenicity, for example, anticonvulsant therapy, and switch to alternatives where possible or counsel the female client about the risks of continuing or stopping therapy where alternatives do not exist. All chronic conditions should be optimally managed before pregnancy attempts are undertaken.
- Prepregnancy counselling (see Appendix 1) should also include information about a healthy lifestyle, as well as the negative impact of smoking, excessive alcohol intake and recreational drug use on both fertility and pregnancy outcomes.
- Women who are 35 years and older should be counselled about the impact of advanced maternal age on fertility, first trimester miscarriage, pregnancy complications and pregnancy outcomes including stillbirth and chromosomal abnormalities, most notably trisomy 21 (Down syndrome).^106^ Prenatal screening for foetal abnormalities, and other higher-level care, may be offered based on maternal age according to routine national antenatal care guidelines.
- All women should have a full obstetric history taken as part of the prepregnancy workup. Any woman with heightened obstetric risk should be referred to, or discussed telephonically with, a specialist obstetrician/gynaecologist, where available, to ensure risks with any future pregnancy can be managed.

### Deferring pregnancy for HIV-positive individuals or couples – Is it ever appropriate?

Ultimately the decision whether or not to achieve pregnancy rests with the client and providers should avoid personal judgements, especially concerning socio-economic or relationship status. However, there are medical circumstances in which a clinician should encourage the couple to consider deferring pregnancy, including the following instances:

- One or both partners are not virally suppressed, not consistently adherent to ART for at least six months or PrEP is not in use.
- There is suspicion of infertility and there are available resources to investigate and manage any underlying condition.
- There are relative medical contraindications to pregnancy.
- There is harmful substance use with motivation to stop use of the substance, allowing time to access rehabilitation support.
- The woman had a recent miscarriage.
- The woman has a CD4+ count below 200 cells/mL and only recently started ART, whereby there is still the possibility of immune reconstitution.

In these instances, clients should be encouraged to attempt pregnancy only once health has been optimised. Where absolute contraindications exist, making pregnancy permanently inadvisable, the couple needs to be counselled accordingly, including the availability of other options such as fostering and adoption.

Pregnancy should be avoided and other options sought when:

- There is evidence or confirmation of infertility.
- There are absolute medical contraindications to pregnancy.
- There is harmful substance use with no intention to stop substance use.

### Expected time to pregnancy and fertility assessment

Trying to achieve pregnancy through condomless sex should only be undertaken if the couple are presumed fertile and expected to have a reasonable chance of achieving pregnancy. In contexts where resources are limited, this can be difficult to accurately assess. Where fertility potential is untested, the couple should try for at least six months before investigations are considered.

HIV itself can negatively impact male and female fertility.^107^ Fertility, although improved with ART, may not return to normal. In the female, HIV may be associated with anovulation, amenorrhoea and premature ovarian failure. In males, HIV may negatively impact testosterone levels, libido and may contribute to erectile dysfunction.^107^

### Expected time to pregnancy

It is recommended that a presumed fertile, HIV-affected couple attempt pregnancy for at least six months before considering possible infertility.^108^ The best chance of pregnancy is seen in the first six months of attempting pregnancy.^109^ Although infertility is defined as the failure to achieve pregnancy after one year of regular, condomless intercourse, HIV-affected couples should be referred sooner, where referral options exist, to avoid prolonged HIV risk exposure. Failure to achieve pregnancy can be a considerable source of relationship stress so the provider should explain to any presumed fertile couple that, depending on age, up to 50% of couples in the general population will achieve pregnancy in the first six months, a further 30% – 40% of couples who continue trying will achieve pregnancy by 12 months and a further 5% – 15% will achieve pregnancy if they continue trying up to 24 months. Up to 5% – 10% of couples will not achieve pregnancy, suggesting underlying infertility.^110,111^

### Pregnancy testing

It is recommended that female clients perform a pregnancy test, using a home testing kit or by consulting with their provider for testing, if they miss their next expected menstruation. Urine beta-human chorionic gonadotropin (BHCG) is a reliable test to confirm pregnancy.

### Suspected infertility

Where infertility is suspected, it is important that couples understand that male and female causes of infertility are equally common and it should never be assumed that one partner is ‘responsible’ for difficulty in conceiving (see Table 5).^112^ In 22% of couples the infertility is male cause only. In 31% it is female only, with secondary infertility being more common than primary. In 21% both partners have an underlying problem. In up to 14% of couples no cause is found and 12% of couples conceive while undergoing infertility investigations.

**TABLE 5:** Causes of female infertility in sub-Saharan Africa.

Female	%
Bilateral tubal disease	41.9
Ovulatory disorders	17.9
No demonstrable cause	13.7
Pelvic adhesions	11.1
Acquired tubal abnormalities	10.3
Hyperprolactinaemia	4.3
Endometriosis	0.9

*Source*: World Health Organization^112^

### Fertility assessment in low-resource settings

A thorough history can detect warning signs of possible underlying fertility issues, including the following:

- previous history of STI
- no history of prior pregnancy despite regular condomless sex
- previous ectopic pregnancy
- possible male factor infertility (e.g. previous vasectomy; undescended testis).

For guidance on how to take a detailed fertility history providers are referred to the following resource:^84^ http://www.fertilitytool.com/tools/basic-tool-4-diagnose-infertility/support-tool-4-how-to-diagnose-infertility/action-1-take-infertility-history/. (Note: The FIGO fertility toolbox is an invaluable tool for providers working in all settings who find themselves managing clients with suspected or confirmed infertility.)

Where referral options for further investigation and management do not exist, couples should be counselled about the risks of trying, or continuing to try, to achieve pregnancy where infertility is suspected. Alternative parenting options or adjusting to the possibility of childlessness should be discussed.

### Fertility assessment in high-resource settings

Where resources are available, clients with a history indicative of infertility, or who have already tried to conceive for more than six months, may be referred to specialist fertility services for further workup, including hormone profiles in women and semen analysis for male partners. Fallopian tube blockage is also a common cause of infertility, which can be readily investigated via a hysterosalpingogram and, if a uterine abnormality such as fibroids is suspected, then a pelvic sonar should be performed. Where a couple is confirmed to be infertile, they may be candidates for assisted reproductive technologies such as IVF, IUI or ICSI, although these are often either unavailable or unaffordable for the majority of people living in high HIV prevalence settings.

### Managing safer pregnancy outcomes

#### When pregnancy is confirmed

- All female clients with confirmed pregnancy should be linked to their local antenatal care provider as soon as possible, preferably on the same day.
- *HIV-positive female*: repeat VL on the day pregnancy is confirmed to ensure sustained viral suppression. Emphasise treatment adherence throughout pregnancy and breastfeeding, providing reassurance about the safety of ART exposure during pregnancy.
- *HIV-negative female*: retest on the day of pregnancy confirmation and after the window period according to the last risk exposure. If the woman is on PrEP, discuss continuing versus returning to other HIV prevention strategies. Regular retesting, at least three-monthly, should be encouraged throughout pregnancy and breastfeeding, and HIV prevention strategies, including consistent condom use, should be encouraged.
- *HIV-positive male partner*: ensure the male partner remains linked to routine ART services. If eligible, he may benefit from joining an adherence club or other form of differentiated care for stable patients.
- *HIV-negative male partner*: retest on the day of pregnancy confirmation and at the end of the window period according to the last risk exposure.
- All couples should be encouraged to return to consistent condom use, not only for HIV transmission prevention but also avoidance of new STIs.
- Provide information about antenatal, intrapartum and postnatal care
  ■ Pregnancy confirmation provides the perfect opportunity to provide basic information about what to expect during the antenatal and postnatal periods in terms of VL monitoring or HIV retesting, management of the baby if they are HIV exposed and recommended infant feeding options.
- Ongoing male partner involvement should be encouraged, and male involvement should be supported by a conducive environment being provided in antenatal and postnatal care services. Male involvement has been shown to improve maternal and infant outcomes, overall and in terms of PMTCT.^113,114^
- Provide information about safe infant feeding to lay a foundation for repeated counselling throughout pregnancy.^115^
- The female (and if possible, her male partner) should be advised about post-partum contraception to avoid future unintended pregnancy and to support appropriate spacing should another child be desired in the future.
- Couples who are engaged in safer conception care often become aware of their pregnancy very early on, sometimes at just 2–3 weeks gestation. Miscarriage remains a significant risk up until around 14 weeks of pregnancy and couples should be counselled about this possibility but reassured that there is little that can be done to prevent miscarriage. If miscarriage does occur, they should return straight to the clinic for appropriate management.
- The woman should be encouraged to remain on folic acid supplementation throughout at least the first three months of her pregnancy. Other antenatal supplements should be given as per local guidelines.

#### Counselling for those experiencing miscarriage

If an individual or couple returns to report a miscarriage, appropriate counselling should be provided and the woman managed as per local guidelines. It is important to acknowledge the disappointment and grief associated with miscarriage and to emphasise to the couple that no one is to blame and that early pregnancy loss is usually a result of an abnormality that occurred during early development of the foetus. Miscarriage is common, occurring in up to 25% of pregnancies. A couple is not at any increased risk of a further miscarriage unless they have had three or more miscarriages, at which point they should be referred to a specialist for further investigations. After a miscarriage, couples may want to start trying to conceive again straight away. It is recommended that the couple can start trying within three months from their loss, if they feel ready to do so.^116^ The preconception workup should be repeated to ensure that the couple are still ‘safe’ to start trying again.

### Special situations

#### What if an HIV-positive woman desires a child but does not have a partner?

Options include insemination from alternative sperm sources such as a sperm bank or known male sperm donor (HIV status should be known), surrogacy or adoption. It is worth knowing what resources exist in your area, what the stipulated eligibilities are and what financial resources would be required.

A man or woman may also present without a stable partner, communicating their desire for a child. Whether they have one or multiple current, but not necessarily long-term, partners, they should still be offered all the available risk reduction strategies without judgement. Counselling should include a discussion about the increased risks associated with attempting to conceive via, or impregnate, multiple partners, if that is their chosen approach to fulfil their fertility intentions.

#### Men who have sex with men, transgender and female same sex couples

Options also exist for couples who may not be able to conceive naturally because they are in a same-sex relationship or one or both partners are transgender. Where necessary, further advice should be sought from the appropriate specialist to support these couples to achieve their reproductive goals as they have the same reproductive rights as heterosexual couples. Options include surrogacy for same-sex male couples and sperm donation for same sex female couples. A transgender male who is in a relationship with a cis-male (Cis-male is a male whose gender identity corresponds to the biological identity assigned at birth) may still be able to conceive and carry the pregnancy to term and should remain engaged in care to appropriately manage hormone therapy throughout pregnancy.

## Figures and Tables

**FIGURE 1-A3:**
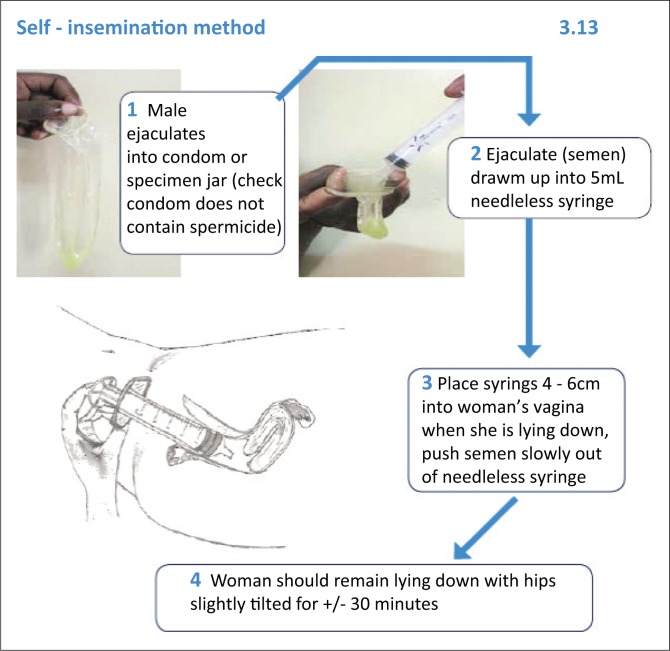
Diagram of self-insemination.

## Appendix 1: Prepregnancy counselling – Checklist and key messages

Prepregnancy counselling should ensure an informed choice about reproductive options, including any risks and costs involved with each strategy, as well as the likely chances of success.

Prepregnancy counselling should include the following:

- A summary of available data on the safety of different strategies, and the impact of combining strategies to optimise risk reduction.
- All safer conception strategies are considered risk reduction strategies and no provider should guarantee a risk-free process.
- The importance of viral suppression for the HIV-positive partner with continued full adherence to ART (undetectable equals uninfectious: U=U) should be clearly explained.
  ■ Because VL cannot be monitored continuously, full adherence is key to this approach, particularly if it is to be used in isolation, without any other strategy.
  ■ Should there be an interruption in ART drug supply, or suboptimal adherence, at any point the client must alert their provider and pregnancy attempts should be deferred until viral suppression can be reconfirmed or other prevention strategies introduced.
- A discussion of PrEP as an option in certain situations, especially where viral suppression may not be guaranteed, for example, HIV-positive partner recently initiated ART or concerns about adherence, not yet engaged in care or of unknown HIV status.
- The importance of regular STI screening.
- The need to assess for reduced fertility at an early stage to avoid the possibility of prolonged risk exposure during ongoing pregnancy attempts in the context of infertility where a couple has not conceived after at least six months of correctly timed pregnancy attempts.
- The risk of transmission to a non-infected partner and MTCT if the female partner is HIV-infected, or becomes HIV-infected, and is not established on ART with an undetectable VL.
- The importance of reporting any pregnancy as soon as possible to ensure early access to antenatal care so that maternal healthcare and PMTCT interventions can be optimally provided throughout the pregnancy.
- Information about available PMTCT strategies for use throughout the pregnancy and breastfeeding period.
- The importance of lifelong adherence to ART for any HIV-positive partner, for their own health and the prevention of horizontal and, for women living with HIV, vertical HIV transmission.
- All female partners, regardless of HIV status, should be started on folic acid supplementation, as per local guidelines, prior to undertaking pregnancy attempts.

Key counselling points about conception options

Prepregnancy counselling should include the presentation of all safer conception options so that the individual or couple can make an informed decision about which strategy, or combination of strategies, they would like to use:

- Condomless intercourse, even without peak fertility timing, is now considered a very safe option in the setting of consistent ART use with confirmed viral suppression (< 200 copies/mL), ongoing full adherence and STI screening for both partners, where possible.
- Where viral suppression cannot be confirmed, explore additional strategies including limiting condomless intercourse to the peak fertile window, PrEP and MMC.
- In resource-intensive settings, explore pros and cons of undertaking more costly conception options, including sperm washing and IUI, IVF or ICSI.

## Appendix 2: Determining a woman’s fertile window

**The basics**: Ensure that clients understand how a woman’s reproductive cycle works. This is often poorly understood and forms the basis for optimising the strategy of timed condomless sex or self-insemination for safer conception.

**Remember**: For those HIV-affected couples who are U=U, precise timing of condomless sex to the fertile window is not considered necessary. If they are comfortable to do so, the couple should be advised to have regular, condomless sex, two or three times per week spread out equally over each week when the woman is not menstruating. For those couples who are anxious about multiple condomless sex acts, the strategy of timing condomless sex to limit them to the days of highest fertility can be explained.

Explain the following:

- When an HIV-affected couple is advised to, or chooses to, limit their condomless sex acts to peak fertility, determining a woman’s fertile period is necessary to establish the timing of periovulatory intercourse. There are various ways in which a woman’s fertile period can be determined. The methods described here presume normal fertility and require minimal resources.
- In situations where a woman’s fertility may be impaired, more resource-intensive methods such as Day 21 progesterone measurements or serial ultrasound monitoring, with or without ovulation stimulation through clomiphene administration, may be used by a reproductive specialist. These more intensive methods may also be used in women living with HIV (who have presumed normal fertility) in order to increase their chance of fertility prediction.

### Fertile dates

The average normal duration of a menstrual cycle is 28 days. The first day of a woman’s menstrual period is considered to be Day 1 of her menstrual cycle. Ovulation is anticipated to occur 14 days before the next period is due to start. Her fertile period would be from five days before predicted ovulation up until 1–2 days after ovulation. For example, in a woman whose cycle is 28 days long, ovulation would be assumed to occur on Day 14. The woman’s fertile period would therefore occur between Days 9 and 16 of her menstrual cycle. This creates a seven-day window. This may be narrowed down to a peak fertile window of four days if the couple would like to minimise risk exposure further. This would be calculated as two days before predicted ovulation, the day of ovulation and one day after predicted ovulation.

However, menstrual cycle length may differ considerably between women and may even differ from month to month for an individual woman. It is therefore essential that a woman keeps a record of her menstrual cycle (typically taking into account the first day of her menstrual period) in order to determine an average menstrual cycle length. It is important to explain to patients that regular menstrual cycles may not necessarily indicate that ovulation has occurred. Ovulation is less likely to be occurring in women whose cycle is 24 days or less or 35 days or longer. There are now several mobile phone applications that are available for free that can assist women to keep track of their cycles and to predict ovulation. This can be a very helpful resource. Examples include CycleBeads™ and Dot™. It may take up to four months to establish an average cycle length where cycles are irregular and the more months that are recorded the more accurate the timing can become when using this method.

### Ovulation prediction kits (for urine and saliva)

A number of over-the-counter products are available that enable ovulation prediction. These methods may utilise sampling and analysis of either urine or saliva and detect the surge of luteinising hormone that occurs immediately before ovulation. These test kits are becoming more affordable and can limit risk exposure to just two days per cycle because of their high levels of accuracy. The use of ovulation prediction kits can be particularly useful for women with irregular cycles where the calendar method is often not as helpful. In women where there may be a concern about anovulatory cycles, ovulation test kits may also help to identify if the luteinising hormone surge is not happening and there is a need for further investigation and management to promote ovulation.

### Cervical mucus monitoring

Monitoring the cervical mucus changes that occur around the time of ovulation is also a useful means to identify peak fertility. During non-fertile days, the cervical mucus is thick and acidic. In contrast, during fertile days, the mucus undergoes a change to become thin, profuse, transparent and ‘stretchy’ (spinnbarkeit), like raw egg white. A woman’s awareness of these changes in her cervical mucus may help her to predict her fertile period and guide her pregnancy attempts. However, this method may not be as easy to use for some women, particularly those who use certain hygiene practices including douching or vaginal drying, as the cervical mucus is disrupted or washed away so changes in vaginal secretions may not be as easily observed. If the provider gathers a history of vaginal hygiene practices, it is advisable to counsel the client against such practices as there may be an association between vaginal douching or drying and increased rates of bacterial vaginosis,^117^ which in turn may be associated with increased rates of HIV transmission and pregnancy loss.^118,119^

## Appendix 3: Low-technology sperm collection and self-insemination techniques

Artificial insemination is the process whereby semen is introduced into the female reproductive tract other than by sexual intercourse. It may be intrauterine or vaginal, the former being a specialist procedure. The latter is a low-risk procedure that can be carried out by a healthcare provider or at home by the patient herself or her male partner.

It is advisable that vaginal insemination be attempted at the most fertile time in the menstrual cycle, as described above.

Semen needs to be provided in a clean receptacle, either by male ejaculation into a condom during intercourse or by male ejaculation into a clean specimen jar provided for the purpose. The semen (most men ejaculate 1.5 mL) should be inseminated as soon as possible, and within an hour of ejaculation. If the ejaculate is collected from a condom the couple must ensure that the condom is one that does not include spermicide and the transfer to syringe or clean jar should be done immediately, as latex condoms can cause sperm immobility if exposure is prolonged.

Other equipment to carry out the vaginal insemination would include a ‘turkey baster’, 5 mL or 10 mL plastic needleless syringe or plastic disposable pipette. These items should be supplied to prospective couples or individuals along with the instructions in the diagram.
